# Age-Related Alterations in Immune Function and Inflammation: Focus on Ischemic Stroke

**DOI:** 10.14336/AD.2023.0721-1

**Published:** 2024-05-07

**Authors:** Qiuxin Chen, Minmin Wu, Qiang Tang, Peiyu Yan, Luwen Zhu

**Affiliations:** ^1^The First Affiliated Hospital of Heilongjiang University of Chinese Medicine, Harbin 150000, China; ^2^Heilongjiang University of Chinese Medicine, Harbin 150000, China; ^3^The Second Affiliated Hospital of Heilongjiang University of Chinese Medicine, Harbin 150000, China; ^4^Faculty of Chinese Medicine, Macau University of Science and Technology, Macau 999078, China; ^5^State Key Laboratory of Quality Research in Chinese Medicines, Macau University of Science and Technology, Macau 999078, China

**Keywords:** stroke, aging, immunosenescence, glial cells, inflammaging, senotherapy

## Abstract

The aging of the global population poses significant scientific challenges. Moreover, the biological process of aging is the most significant risk factor for most chronic illnesses; therefore, understanding the molecular and cellular mechanisms underlying these aging-related challenges is crucial for extending the healthy lifespan of older individuals. Preventing brain aging remains a priority public health goal, and integrative and comprehensive aging analyses have revealed that immunosenescence is a potential cause of age-related brain damage and disease (e.g., stroke). Importantly, the neuroinflammatory and immune systems present two-way contact and thus can affect each other. Emerging evidence supports the numerous effects of immunosenescence- and inflammation-mediated immunity in neurologically injured brains. In this study, we briefly outline how aging alters the pathophysiology and transcriptional amplitude in patients who experienced stroke and then discuss how the immune system and its cellular components and molecular mechanisms are affected by age after stroke. Finally, we highlight emerging interventions with the potential to slow down or reduce aging and prevent stroke onset.

## 1. Introduction

Stroke is the leading cause of mortality and disability [1]. Most cases are ischemic stroke, in which blood flow to the brain is reduced or interrupted by arterial occlusion, which is usually caused by emboli originating from the heart or large arteries, localized thrombosis, arterial dissection, or vasculitis [2]. Ischemic stroke therapy aims to promptly restore cerebral blood flow; however, few patients are eligible for this treatment [3]. Numerous studies have demonstrated that when cerebral blood flow is interrupted, a cascade of detrimental events occurs in the brain, including neurotoxicity, microvascular thrombosis, and inflammation, resulting in ischemic stroke outcomes irrespective of reperfusion treatment [4]. Moreover, 80% of strokes occur in people over 65 years of age, and the incidence of stroke doubles every decade after the age of 55. In addition to the higher incidence observed in older patients, they also present higher mortality rates, experience more severe deficits, and recover more slowly than younger patients [5].

Age-related degeneration of the central nervous system (CNS) depends on various mechanisms associated with chronic inflammation and a subsequent decrease in the immune response [6]. Additionally, dysregulation of molecular mechanisms of the immune system with aging accelerates neurodegeneration and plays a vital role in determining outcomes, including for stroke. Immunosenescence and inflammation are subclinical processes that underlie age-related conditions [7]. Immunosenescence is a condition in which the immune system deteriorates, and the immune response becomes dysregulated due to cellular senescence; thus, older persons are more vulnerable to the associated effects [8]. Although inflammation effectively eliminates pathogenic microorganisms and toxic substances from the body, the low-grade chronic inflammation (inflammaging) associated with aging increases the risk of stroke [9]. Inflammaging is characterized by elevated blood levels of acute-phase proteins (e.g., C-reactive protein) and proinflammatory substances (e.g., tumor necrosis factor-alpha [TNF-α], interleukin-1β [IL-1β], and IL-6) [10]. This suggests that the balance between pro- and anti-inflammatory molecules is lost with age and that the inflammatory state predominates, resulting in various levels of damage to the entire organism [9]. An in-depth investigation of immunosenescence and inflammaging at the cellular and molecular levels may aid in clinical decision-making and improve the prognosis of older patients who experienced stroke. Therefore, the effects of aging on other known biological systems, such as the immune system, should be further investigated to comprehensively understand the risk factors, pathology, and recovery process of stroke (Table 1).

Here, we summarize the recent advancements in aging-induced dysregulation of the immune system in stroke and changes in transcriptional amplitude, with an emphasis on functional analyses of aging immune cells and their potential impact on stroke. In addition, we briefly discuss the molecular mechanisms and biomarkers of stroke-related cellular senescence, including telomere attrition, DNA repair, genomic instability, mitochondrial dysfunction, epigenetic alterations, and impaired autophagy. Finally, we discuss preventive or therapeutic ideas that may delay or reduce aging and thereby prevent stroke onset.

**Table 1:** Age-related alterations in innate and adaptive immune system cell types in stroke.

Cell type	Role in the immune system	Senescence related alterations	Implications	Ref.
Microglia	Clearance of debris, damaged neurons, and protein aggregations; synaptic development/regulation; cytokines release; repair injuries; and phagocytose foreign body	Proinflammatory cytokines (IL-1β, IL-6, and TNF-α), ROS, NO, IFN-I signaling, lipid droplets, and exosomes (C1q, C3a, and C3b) ▲ Homeostatic genes, motility, phagocytic capacity, synaptic function, angiogenesis, and transcriptional plasticity ▼	Compared to young mice, aged microglia cause an increase in the number of brain-infiltrating immune cells and an increase in infarct size in older mice.	[5, 11, 12]
Astrocyte	Providing growth factors, counteracting oxidative stress, and producing functional extracellular mitochondria to support neuronal viability	GFAP, glial scarring, neurotoxic saturated lipids, astrocyte reactivity, CXCL10, CXCL5, MHC-1 ▲ Cholesterol synthesis, and glutamate uptake ▼	The function of astrocytes to promote neuroprotection deteriorates with age. Moreover, M1 microglia induce neurotoxicity and neuroinflammation, which stimulates the conversion of astrocytes into neurotoxic-reactive astrocytes A1.	[13]
Neutrophils	Recognition, phagocytosis, and destruction of pathogens	Apoptosis process, ROS, MMP-9, NETs, metabolically active, and augmented production of enzymes responsible for vascular remodeling and BBB permeability ▲ Phagocytic ability, adhesion, clearance of debris, degranulation of antimicrobial proteins, TLR signaling, and chemotaxis ▼	Aged mice with brain-infiltrating neutrophils have been linked to a higher risk of morbidity and mortality in patients with ischemic stroke.	[14, 15]
Macrophages/Monocytes	Activation of the immune response acts as a "pathogenic sensor."	Angiogenesis, tissue remodeling, and circulating cells ▲ Phagocytic ability, phagosomal ability, chemotaxis, and migratory ability ▼	Splenectomy restores peripheral inflammatory cell levels and reduces infiltration of peripheral immune cells into the brain, as the spleen is a source of MDMs in the brain and is critical for regulating the immune response after stroke.	[16, 17]
Natural Killer cells	Recruit neutrophils and macrophages and activate DCs, as well as T and B lymphocytes.	The number of NK cells, CD56dim, CD57, CD16 ▲ NKCC, telomere length, perforin degranulation expression, CD56bright, and the production of cytokines and chemokines▼	Through changes in TLR function and subtype shifts, NK cells become overactive with age and play a role in the inflammatory response.	[18, 19]
Dendritic cells	DCs are the most effective APCs; they have an important interaction with adaptive immunity.	proinflammatory cytokines, autoantigens ▲ MHC-II, I3K signaling pathway, TLR signaling, phagocytic ability, and antigen migration and uptake ▼	Increased risk of infections and autoimmune disease.	[17, 20]
T lymphocytes	The thymus is pivotal for the maturation and development of a diverse T-cell population. Aid in inducing an adequate response to pathogens and neoplasms.	ROS, p38 MAP kinase, Th17/Treg cells, memory CD8+ T cells, memory cells, and CD28- ▲ TCR repertoire, diversity, proliferative capacity, naive cells, CD40L, and telomeres. Telomerase activity ▼	Age-related thymic atrophy, memory conversion, shrinkage of the TCR pool, and increased migration to non-lymphoid tissues. Increased susceptibility to infectious processes.	[17, 21, 22]
B lymphocytes	Antibody production	ABCs, memory cells, TNF-α, and NF-κB signaling pathway ▲ Progenitor B cells, BCR repertoire, naïve cells, expression of costimulatory molecules (CD40), high-affinity protective antibodies, and vaccination ▼	ABCs biomarkers are associated with decreased class-switch recombination and somatic hypermutation of immunoglobulin genes. Increase the risk of infection in elderly individuals.	[23, 24]

IFN-I, type I interferon; ROS, reactive oxygen species; NO, nitric oxide; GFAP, glial fibrillary acidic protein; MHC-I, histocompatibility complex I; NETs, neutrophil extracellular traps; MMP, matrix metalloproteinase; BBB, blood-brain barrier; MDMs, monocyte-derived macrophages; NKCC, NK cell cytotoxicity; NK, natural Killer; DCs, dendritic cells; APCs, antigen-presenting cells; TCR, T-cell receptor; ABCs, age-related B cells; ▲, raise; ▼, decrease.

## 2. Role of Aging in the Pathophysiology of Stroke

Risk factors for stroke include sex, age, diet, lifestyle, smoking, and glucose abnormalities, and age is the most potent and reliable [25]. Age-related increases in stroke risk have been observed in both men and women, particularly those over the age of 55 [26]. Youth enhances resistance to ischemia and positively affects recovery after ischemic damage [27]. Younger patients who experienced stroke have a better functional prognosis when adjusted for stroke severity at baseline, whereas older patients have an increased risk of stroke and worsened functional outcomes, including immune system deficiencies, vascular dysfunction, and increased risk of infection [27, 28]. Notably, aging is another adverse prognostic factor for ischemic brain tissue, and ischemic but viable tissue is more likely to develop into infarction in the penumbra region and the overall size of ischemic lesions are more likely to increase in the elderly population [29]. Advances in penumbra imaging may enable the noninvasive preservation of salvageable tissue. Recent studies have demonstrated that neuroimmune-targeted therapy is an effective strategy because the therapeutic window of expected efficacy is extended, and fewer hemorrhagic complications are observed [30].

Similar to the nervous system, the immune system also undergoes aging. These age-related changes determine susceptibility to infectious disease, elevate the risk of cancer and cardio-cerebrovascular disease, and decrease vaccination efficacy [31, 32]. The senescence cascade response of the immune system may exacerbate the alterations caused by brain aging and age-related neurological damage or disease [33]. Specifically, the architecture of lymphoid organs, where immune cells mature, develop, and reside, and the composition and function of cell subsets change with age [34]. Immunosenescence, which amplifies neuroinflammation, progressively alters and deteriorates the immune system over time and eventually causes negative consequences in older individuals, such as stroke [35].

The three main barriers in a healthy brain that protect the parenchyma from foreign pathogens are the blood-brain barrier (BBB), choroid plexus (CP), and meninges. Under normal environmental conditions, immune cells circulate freely in the blood and a few lymphocytes perform immune surveillance in the cerebrospinal fluid (CSF). The disrupted environment after ischemic stroke triggers a series of pathological cascade responses through the immune system and neuroinflammation. However, aging further exacerbates these responses, as detailed in Section IV.

**Figure 1.**
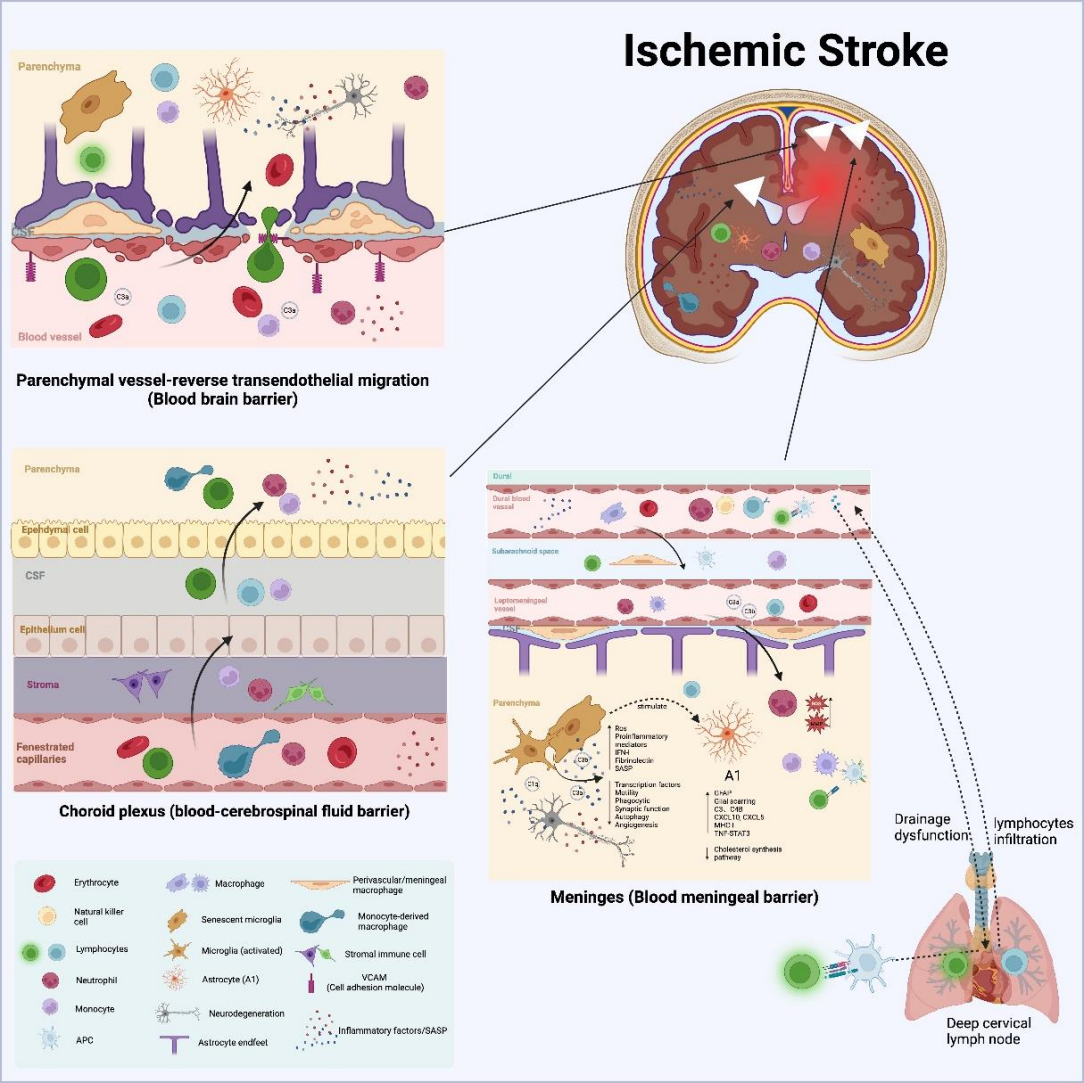
**Age-related changes in cerebral ischemia stroke**. Aged microglia became activated, upregulated phagocytic phenotype and proinflammatory factors, reduced the proportion of microglia M1/M2, showed more downregulated genes and less upregulated genes, and IFN-I may be involved in the transcriptional response of genes. Disruption of the BBB and loss of tight junction proteins enable leukocytes to roll and adhere to the luminal side of the vessel and subsequently move from the vascular lumen to the brain parenchyma. Moreover, leukocytes are transferred to the brain parenchyma through the blood-membrane and blood-cerebrospinal fluid barriers. C1q, C3a, and C3b hyperactivate microglia phagocytosis and exacerbate synaptic function through a C3aR-dependent mechanism. Aged microglia stimulate the conversion of astrocytes into neurotoxic responsive astrocytes A1. IFN-I, induced type-I interferon; BBB, blood-brain barrier

The BBB, which protects the brain from pathogens and restricts the entry of circulating substances, primarily consists of brain endothelial cells (BECs). The aging phenotype of BECs develops in the brain microvasculature and includes microvascular thinning, proinflammatory changes, vasodilatory dysfunction, and BBB disruption [36]. Senescent BECs promote neurovascular uncoupling by deregulating angiogenesis and vascular endothelial growth factor (VEGF) or by increasing the reactive oxygen species (ROS)/reduced nitric oxide (NO) axis [37]. Dysregulation of neurovascular coupling leads to a loss of astrocyte endfoot coverage, disrupts communication between neurons and endothelial cells, and exacerbates neurodegeneration [37]. In age-related cerebrovascular diseases, proinflammatory senescence-associated secretory phenotype (SASP) mediators (IL-6 and IL-1β) induce a leaky BBB, which contributes to peripheral immune cell infiltration [38]. Senescent plasma upregulates vascular cell adhesion molecule 1 (VCAM1) in BECs, which boosts the capacity of immune cells to infiltrate the CNS in response to vascular damage, thereby impairing neural precursor cell activity and increasing microglial reactivity (Fig. 1) [39]. For example, Chen et al. found that the infusion of young plasma into aged mice produced a rejuvenating effect on BECs [40]. Additionally, short-term infusion of aged plasma into young mice upregulated innate immune and oxidative stress response pathways [40]. These results further confirm that BECs in the BBB are a possible target for treating age-related diseases.

The CP is an epithelial monolayer that forms the blood-CSF barrier and produces CSF. After ischemic stroke, monocyte-derived macrophages (MDMs) enter the CSF via the CP (Fig. 1) [41]. Aging-induced type-I interferon (IFN-I) signaling in the CP impairs brain function [42]. Although IFN-I was initially identified because of its antiviral properties, it has a vital role in reducing inflammation within and outside the CNS. CP and BECs are important factors in preventing or promoting peripheral inflammatory signals, such as IFN-1 and MDMs.

Neutrophils have been found in the extraluminal sites of soft meningeal vessels within hours after stroke in mice and humans, suggesting a strong correlation between neutrophils and soft meningeal vessels (Fig. 1) [43, 44]. Age-related meningeal lymphatic vessel (MLV) dysfunction has also been observed in aged mice [45]. Specifically, CSF perfusion is impaired in aged mice, which is accompanied by a reduction in the coverage and diameter of the MLVs and a decrease in CSF drainage to the deep cervical lymph nodes. Meningeal lymphatic endothelial cell (LEC) gene expression analysis also confirmed significant downregulation of lymphangiogenic growth factor signaling in LEC during aging [45]. Using magnetic resonance imaging, Zhou et al. identified a strong relationship among the glymphatic pathway, MLV, and aging [46] and showed that the transport capacity of the glymphatic pathway and MLV is significantly impaired in the aging human brain, which hinders the clearance of senescent cells and interstitial waste. They also found that MLVs were located downstream of the glymphatic route.

Although the leukocyte immune response in the brain parenchyma has been clarified, whether meningeal neutrophils continue to infiltrate areas of ischemia has not been clarified and how inflammatory responses change in the meninges of the ischemic aging brain has not been revealed [47]. Therefore, future studies should precisely subcategorize the individual meningeal layers to assess their unique structural and functional properties separately to identify and clarify the utility of immune cells associated with the aging brain and boundary compartments (meninges) during stroke. Moreover, new therapeutic strategies targeting age-related MLVs, and the intracerebral lymphatic system may aid in delaying the onset and progression of stroke [48].

## 3. Aging Alters the Magnitude of Transcription Post-Stroke

Although gene expression studies in animals with stroke using microarrays and RNA sequencing (scRNA-seq) have provided great insights into the pathophysiology of ischemic stroke [49, 50], only a few studies have examined gene expression in aging animals with stroke.

Androvic et al. investigated the effects of ischemia in aged mice and identified 400 upregulated genes that increase the neuroinflammatory milieu. This upregulation is followed by the secretion of proinflammatory cytokines, activation and infiltration of peripheral immune cells, and activation of signaling pathways (IFN-I, ERK/MAPK, and cAMP/cGMP) that contribute to secondary injury [5]. Moreover, aged mice exhibit gene downregulation after stroke, thus leading to the dysregulation of axonal and synaptic maintenance genetic programs, which affects functional recovery after stroke [5]. IFN levels have been proposed to correlate with the severity of the injury, with low levels beneficial for mild ischemic preconditioning and high levels detrimental for severe stroke. This finding is consistent with the fact that older animals, which present greater gene upregulation, generally experience more severe strokes.

Bolte et al. studied the transcriptional response of meninges to traumatic brain injury (TBI) in young and aging mice using single-cell RNA sequencing (scRNA-seq) and showed that TBI leads to the upregulation of IFN-I signature genes in aged meningeal macrophages and controls the upregulation of inflammation-related genes in fibroblasts and adaptive immune cell populations [51]. These findings revealed that ischemic stroke and aging have an effect on the meningeal transcriptome. Further transcriptomic analysis confirmed that an aging immune system might increase the risk of potentially harmful proinflammatory and autoreactive responses after stroke [52]. Recent studies have used RNA-Seq to analyze the transcriptome of human neural stem cells (hNSC) after transplantation into aged mice with stroke. The expression of genes encoding TNF receptors (Tnfrsf1b, Tnfrsf1a, and Tnfrsf10b), adhesion and leukocyte extravasation molecules (Esam, Icam1, Icam2, Mcam1, and Pecam1), apoptotic factors (Casp8 and Fas), proinflammatory factors (Il6ra, Il1r1, Ccl2, and Il6), and Toll-like receptors (TLRs) (Tlr1, Tlr13, Tlr7, and Tlr8) were downregulated in the brains of aged stroke mice after hNSC transplantation compared to those without hNSC transplantation. Furthermore, hNSC transplantation reduces infarct size and proinflammatory factor (TNF-α, IL-6, and IL-1β), matrix metalloproteinase-9 (MMP-9), and MMP-3 expression in aged stroke mice [53].

Transcriptomic data are usually interpreted via correlation, and over-interpreting the results should be avoided. To establish a causal relationship between changes in expression and altered immune cell function in animal studies of aging stroke, further mechanistic studies must be performed and validated in vivo. Multi-omics and single-cell technologies will lead to a better understanding of the transcriptional and cellular changes in aging-associated neurodegenerative diseases (Table 2) [5].

**Table 2:** Aging alters the magnitude of the transcriptional response to ischemic stroke.

Functions	Differentially expressed genes/ Transcription activity	Ref.
Astrocyte activation	Plin4, Gfap, Anln, Pcdhb6, Serpina3n, Lyz2, Neat1, and C4b ▲	[54, 55]
Microglia activation	Clec7a, Cst7, Cybb, C4b, Ccl8, Lgals3, Mmp12, and Spp1 ▲ Cxcr2, S100a8, Il1b, and Mmp9 ▲	[56]
Oligodendrocyte precursor cells activation	Rab37 and Tnfaip2 ▲	[57].
Neutrophil	Aif1, Ly86, and Il1b ▲ Ly6g ▼	[58]
MM1	Apoe, Arg1, Ym1 (Chil3), Cd93, Hmox1, Tgfbi, and Hif1a ▲	[58]
MM2	Cybb, Il1b, C3, and Hp ▲	[58]
DC	H2-Aa, H2-Ab1, and Cd74 ▲	[58]
T cell-mediated immunity	B2m, Rsad2, Arg1, Il1b, Sash3, and MHC I ▲	[5]
T cell activation, cell adhesion	Runx3, Lgals9, Sash3, Card11, Il15, Cd28, and Cd5 ▲	[5]
B cell activation	Bst1, Card11, Cd38, and Mzb1 ▲	[5]
Phagocytosis	Gulp1, Rab20, Srpx, Igha, Ighm, Cd36, and Fcgr2b ▲	[5]
Endothelial cell differentiation	Notch4 and Cdh5 ▲	[5]
Type I interferons (IFN-I) signaling	Stat1, Lrf9, Rsad2, Mx2, Oas3, Usp18, Ddx58, Isg15, Ifi203, Ifi204, Ifit1, Ifit2, Ifit3, Gbp2, and Gbp3 ▲	[5]
Angiogenesis	Anxa1, Adamts9, Ceacam1, Gata2, Adgra2, Adgrg3, and Ankrd1; ▲ Angpt2 and Vegfa ▼	[5, 59]
“Inflammatory” genes	Slc11a1, St14, Tep1, Trem2, Ptprc, Rab32, and Tyrobp; ▲ Cxcl12, Mmp8, Mmp12, Mmp14, Mpeg1, Tnfrsf1a, and Tnfrsf1b ▲	[60, 61]
Inflammasome	AIM2 ▲	[62]
Cell cycle regulation	Ccna2, Ccnb1, Cdk1, Psmb8, and Psme2 ▲	[62]
The regulation of immune response	Arg1, Il7r, Fcgr2b, Nod2, Sh2d1b1, Ceacam1, and Cd274 ▲	[5]
The buildup of the fibrotic scar	Cthrc1, Il6ra, Il13ar1, Il18, Mmp2, Rassf4, Tgfb1, Tgfbr2, and Timp1 ▲	[61]
Cytokine secretion	Gsdmd, Trim16, Ddx58, Ifih1, Clec5a, and Nod2 ▲	[5]
Synaptic communication proteins	Cplx1, Syt2, Rims1, Nrxn3, Lin7a, Braf, Cadps, Chrm2, Pnkd, Dnm1, Myo5c, Smpd3, Rab37, Vps41, and Shroom2 ▼	[5]

Compared with young animals, aged animals exhibit a more significant upregulation of ischemic effects on adversely affected genes. Higher infiltration and activation of peripheral immune cells, proinflammatory cytokine secretion, and signaling pathway activation (IFN-I) lead to an increased neuroinflammatory environment and secondary damage in older animals. Moreover, some genes are downregulated in aged animals after stroke, and synaptic communication proteins are specifically repressed. MM, Monocyte/Macrophage; DC, Dendritic cell; ▲, raise; ▼, decrease.

## 4. Aging-Related Immune System Alterations in Stroke

During the initial stages of ischemic injury (acute phase), the brain rapidly undergoes impairment of the ion pump (energy failure), disruption of membrane integrity, excessive accumulation of sodium and calcium ions, and necrosis and death of neuronal cells. Immune responses are initiated after cellular injury, and damage-associated molecular patterns (DAMPs; e.g., high-mobility group box 1 and heat shock proteins 60) and cytokines (ROS and proinflammatory substances) produced by injured and dying neuronal cells are diverted through the disrupted BBB or CSF lymphatic pathway to the systemic circulation (Fig. 1) [44]. The lymphoid organs are affected by circulating DAMPs and cytokines that cause inflammatory reactions. DAMPs can directly recruit immune cells by interacting with pattern recognition receptors (PRRs) in the periphery. PRRs recognize the released self-molecules (DAMPs) and non-self viral and bacterial products (pathogen-associated molecular patterns, PAMPs). Moreover, innate immune components use PRRs to recognize and alert organisms to attacks by microbes, transformed cells, or damaged cells. PRRs include TLRs, NOD-like receptors (NLRs), and RIG-I-like receptors (RLRs). TLRs act through intracellular signaling pathways, thus leading to the activation of nuclear factor kappa B (NF-κB) and production of various mediators, whereas NLRs can stimulate the inflammasome complex, thus leading to the production of IL-1β and IL-18. RLRs act through IFN-responsive components. Adaptive immunity encompasses humoral and cellular immunity, and this system recognizes and responds to specific pathogens. Moreover, T and B cells, which are among the most cells in the adaptive immune system, infiltrate the brain differently and persist for weeks or months at 1-3 days after ischemia [63].

The number of both PAMPs and DAMPs increases with age. Aging brains show worse functional outcomes and significant increases in activated microglia, neutrophils, dendritic cells (DCs), and inflammatory macrophages, which can lead to greater behavioral deficits and mortality. Tsai et al. analyzed the short- and long-term peripheral immune responses in patients (63.3 ± 17.4 years) after stroke using mass spectrometry flow cytometry [64]. In the acute phase (day 2 after stroke), the rapid activation of pSTAT3 (increased IL-6 levels) in innate immune cell subsets was associated with poorer cognitive performance. In the chronic phase (day 90 after stroke), several sustained immune responses were observed, including a higher frequency of neutrophils and active Th1 cells and DCs. Furthermore, reducing the circulation of lymphocyte invasion by blocking anti-CD49d, which is a key adhesion molecule, improved functional outcomes in patients who experienced stroke in the acute phase but failed to show efficacy in the chronic phase [65]. Consistent with this, Heindl et al. validated the long-term accumulation and local aggregation of T lymphocytes during the chronic phase of stroke in human and animal brains [66]. Moreover, in a 1-year follow-up assessment of blood biomarkers in patients who experienced stroke (64.5 ± 9.5 years), higher TNF-α and IL-1β levels were associated with poor outcomes at 1-year post-stroke [67].

Notably, converting healthy immune cells to an alternative phenotype during the chronic phase of stroke may provide neuroprotective effects by regressing inflammation. This process mainly involves eliminating dead cells, forming an anti-inflammatory environment, and generating favorable factors that promote tissue repair, thereby partially reestablishing neurological function in patients who experienced stroke. However, in the microenvironment of the pathological state, senescence, chronic immunosenescence and inflammaging impair neurogenesis and functional recovery in elderly patients who experienced stroke through the persistent and unresolved production of proinflammatory mediators, impaired recruitment and infiltration of leukocytes, and delays in inflammatory regression, which lead to further neurodegeneration and tissue loss [28]. Next, we examined how aging influences the pathological effects of ischemic brain injury, including identifying multicellular interactions that may determine the evolution of BBB damage, neuronal cell death, glial responses, and immune cell infiltration (Fig. 1, Table 1).

### 4.1 Brain-resident immune cells

#### 4.1.1 Microglia

The ischemic brain is negatively affected by classically activated M1 microglia/macrophages, which are proinflammatory, whereas alternatively activated M2 microglia/macrophages are anti-inflammatory and protective. In the proinflammatory (M1) state, activated microglia produce several proinflammatory factors, including TNF-α, IL-1β, IL-6, CC-chemokine ligand 2 (CCL2), CCL12, CXCL10, interferon-gamma (IFN-γ), ROS, and NO [68]. Transcription factors activated by M1 microglia trigger the upregulation of cell surface markers, such as histocompatibility complex II (MHC II) and the cluster of differentiation 86 (CD86). M1 microglia induce neurotoxicity, neuroinflammation, and oxidative stress, which stimulate the conversion of astrocytes into neurotoxic-reactive astrocyte A1 [13]. In the anti-inflammatory (M2) state, activated microglia express cell surface markers, such as mannose receptors, Arginase-1, transglutaminase-2, CD206, and CD163, as well as anti-inflammatory molecules, including tumor growth factor-β (TGF-β), IL-4, IL-10, CCL17, CCL22, and neurotrophic factors [69]. Moreover, M2 microglia inhibit M1 microglial function and enhance tissue repair and remodeling by promoting debris removal, angiogenesis, extracellular matrix deposition, axonal growth, and functional synaptogenesis [70]. Studies have examined the polarization response of aged microglia in stroke animal models and showed that in stroke mice, inflammatory phenotypic (M1 and M2) markers present a reduced M1/M2 ratio in microglia in aged mice compared to young mice, indicating a shift toward a proinflammatory state with age after stroke [71].

Aging leads to profound changes in the immune system, including the activation and dysfunction of microglia in the brain. Specifically, microglia activation undergoes age-related changes in migration and phagocytosis that lead to increased neuroinflammation, white matter damage, and cognitive impairment in older adults and rodents [72, 73]. Additionally, aging microglia shift to a proinflammatory state and release TNF-α to upregulate the expression of venous BEC-anchored adhesion molecules (VCAM1 and ICAM1), thereby promoting the transendothelial migration of peripheral T cells [74]. For example, Elmore et al. demonstrated this shift using a colony-stimulating factor 1 receptor inhibitor to noninvasively eliminate senescent microglia, discontinue the drug, restimulate the proliferation of new microglia in the senescent brain, and reverse cognitive, synaptic, and neuronal deficits in aging mice [75]. Recent investigations have shown that aging brains accumulate lipid droplets in the microglia after stroke, possibly due to impaired phagocytosis, exacerbated innate immune responses, and increased expression of certain lipid droplet-associated proteins [76, 77]. Defective phagocytosis results in the excessive production of ROS and proinflammatory cytokines [78]. Studies in rodent stroke models have indicated that enhanced microglial phagocytosis and lipid metabolism contribute to removing lipid-rich tissue debris after stroke, which is vital for functional recovery [47, 79]. Lipid droplet-accumulating microglia (LDAMs) are believed to play a critical role in age-related neuroinflammation and structural and functional impairments in the aging brain. However, future pharmacological and genetic ablation approaches targeting LDAMs may help reduce neuroinflammation and restore homeostasis in the aging brain after stroke.

Transcriptome research has enabled researchers to describe general alterations in aging-related gene expression. Aged microglia are less responsive to ischemic injury than young microglia. Specifically, Shi et al. showed that aged microglia had only 18 upregulated genes and 40 downregulated genes at 3-5 days after middle cerebral artery occlusion (MCAO), whereas young microglia had 250 upregulated genes and 21 downregulated genes [11]. After ischemic stroke, the gene cluster of aged microglia is not activated, which is strongly associated with brain tissue remodeling, functional recovery [5, 11], cell-cell interactions, immune-cell chemotaxis, and immune-inflammatory responses. Similarly, recent studies have revealed transcriptomic differences between young and old microglia during the post-stroke recovery phase (14 days post-stroke), with young microglia also showing a strong trend toward the upregulation of gene expression after stroke compared to that observed in aged microglia [80]. Additionally, reduced motility and impaired activation of the pro-angiogenic cascade in aged microglia may impair angiogenesis in the aged brain [80].

Zhang et al. showed that the levels of proinflammatory mediators (C1q, C3a, and C3b) in serum exosomes increase with age and are associated with worsening stroke outcomes (cognitive impairment and motor deficits) through a C3aR-dependent mechanism that crosses the BBB to initiate and hyperactivate microglial phagoptosis (primary phagocytosis) [81]. Moreover, replacing exosomes from aged rats with those from young rats improved short- and long-term functional recovery and reduced synaptic loss after stroke [81]. Neuronal dendritic trees degenerate with age, and ischemic stroke exacerbates this degeneration. Synaptic organization and functional recovery are vital for the recovery of sensorimotor and cognitive functions in aged animals following ischemic stroke. Therefore, modulating the peripheral and central immune systems through complement-microglial interactions may be a promising therapeutic tool for treating ischemic stroke.

#### 4.1.2 Astrocyte

Astrocytes play an excellent neuroprotective role during ischemia by providing growth factors, scavenging glutamate, maintaining BBB function, counteracting oxidative stress, and producing functional extracellular mitochondria that support neuronal viability [82]. During the acute phase of a stroke, damaged cells in the lesion and penumbra release cytokines. Simultaneously, reactive astrocytes proliferate and form glial scars that maintain CNS homeostasis *in vivo*, isolate lesions, and limit neuroinflammation [83].

However, the neuroprotective ability of astrocytes decreases with age. Clarke et al. found that the response of astrocytes to lipopolysaccharide (LPS)-induced neuroinflammation was correlated with age-related changes. Specifically, older mice have increased numbers of CXCL10-expressing astrocytes that recruit more T lymphocytes, which exacerbate neuroinflammation following neurodegenerative disease injury [54]. Additionally, IL-15-producing astrocytes were found in the brains of patients with acute ischemic stroke (82 ± 9 years) and aged mice [84]. Long-term IL-15 exposure increases the cytotoxicity of T lymphocytes (CD 8+ T cells) toward target cells, which is believed to be the mechanism underlying tissue damage [84]. An increase in A1-like astrocytes with age may play an essential role in enhancing the susceptibility of aging brains to neurodegeneration [54]. Further analysis of A1-like reactive astrocyte gene expression revealed an aging-induced inflammatory response and upregulation of astrocyte-responsive genes [85]. For example, glial fibrillary acidic protein and Serpin3n were upregulated, C3 and C4B were enhanced due to complement system activation, cytokine (CXCL10 and CXCL5) production was increased, and MHC I was upregulated, which exacerbated neurodegeneration and BBB damage in the aging brain. Kim et al. verified that an inflammatory reactive astrocyte state leads to BBB disruption through activation of the TNF-STAT3 signaling axis and secretion of alpha 1-antichymotrypsin [86]. Interestingly, age-related BBB dysfunction has been shown to cause excessive activation of TGF-β signaling in astrocytes, thereby triggering a proinflammatory phenotype [87]. As astrocytes age, they drive neurological dysfunction through loss of function and the SASP, which manifests as neuronal hyperexcitability, neuroinflammation, neurotoxicity, and neurodegeneration. These neurological deficits lead to hippocampal and cortical neural hyperexcitability and cognitive deficits. However, Mendelian randomization may be required to verify whether age-related BBB impairment and aging astrocyte dysfunction are bidirectional.

Selective cerebral hypothermia reportedly attenuates ischemia-induced infarct volume loss and brain atrophy in aged mice and ameliorates short- and long-term white matter damage [88]. This protective effect may regulate astrocyte function by modulating inflammatory and apoptotic factor expression, reducing glutamate release and ROS, decreasing metabolic rate, and preventing BBB destruction [85]. Moreover, Li et al. found that activating the VEGF-C/CCL21 pathway in aging mice contributes to removing senescent astrocytes from the brain via the meningeal lymphatic route [89]. However, many unanswered questions remain regarding the contribution of astrocytes to the selective vulnerability of aging rodents and humans. Therefore, elucidating the functional heterogeneity and molecular mechanisms of aging astrocytes will help elucidate age-related neuro-degenerative diseases. Moreover, such work could help identify novel therapeutic targets for preventing or delaying the aging process.

The results presented herein suggest that brain aging leads to subtle changes in the neuroinflammatory milieu, particularly in neuroglia. Moreover, proinflammatory activation of microglia or astrocytes may result in an unfavorable environment, thereby worsening ischemic damage in aged animals. Transcriptomic and epigenetic studies have reported that glia-specific gene expression patterns change significantly with aging [90]. These reports provide strong evidence to support future explorations of the functional role of aging glial cell genes in the neural circuitry of patients who experienced stroke.

### 4.2 Peripheral immune cells

#### 4.2.1 Neutrophils

Immunosenescence affects neutrophils in elderly individuals. Studies have shown that phagocytosis, ROS release, antimicrobial protein degranulation, and neutrophil extracellular traps (NETs) are defective in elderly neutrophils.

Hematogenous myeloid cells infiltrate the brain after ischemic stroke and are highly activated by resident immune cells [91]. Neutrophils are one of the first leukocyte subsets to enter ischemic tissues, and accumulating evidence indicates that the development of secondary infarcts has a significant detrimental effect on neutrophils [92]. Clinical trials targeting neutrophil-related pathological mechanisms have failed to improve the aging-related dysfunction in patients who experienced stroke. The use of young stroke animals without complications has been identified as a potential reason for this unsuccessful clinical translation [93]. Therefore, an in-depth understanding of the relationship between neutrophil activation and aging is essential for mitigating and addressing these disorders.

Aged animals show significant post-stroke neutrophil infiltration in the brain [14]. This may be associated with age-related microglial deficiency or dysfunction, which enhances neutrophil accumulation in the core of ischemic lesions [94]. Compared to young neutrophils, senescent neutrophils in the ischemic brain exhibit defects when clearing debris and produce excess ROS and MMP-9 [14]. With aging, the balance between the extracellular matrix and MMP is disrupted, which alters the abnormal degradation of extracellular matrix and accumulation of elastin and collagen fibrils, which are hallmarks of vascular aging [95]. The activation of MMPs exacerbates the inflammatory response, disrupts the BBB, and worsens the infiltration of leukocytes, which may lead to cerebral edema and hemorrhage and a worse prognosis after stroke in older patients [15, 96]. Older patients who experienced stroke (>71 years) had more pronounced hyperemia and hemorrhage than younger patients, and neutrophil infiltration was found in the brain parenchyma (areas of hemorrhage and congestion), thus reflecting increased MMP-9 levels [97] and BBB disruption [98]. Higher neutrophil counts before thrombolysis in patients with cerebral ischemia are associated with symptomatic cerebral hemorrhage and a poorer prognosis at 3 months [99]. Moreover, older patients who experienced stroke exhibit altered circulating levels of neutrophil-associated cytokines, with significantly elevated IL-6 and IL-8 levels [97].

Recent studies have shown that adropin, a peptide encoded by an energy homeostasis-related gene, reduces MMP-9 levels and preserves tight junction proteins in aged mice after ischemic stroke [100]. Similar rejuvenating effects can be achieved through splenectomy, young blood transfusion, and young bone marrow transplantation [101]. Aged mice receiving young bone marrow showed reduced post-stroke behavioral deficits, fewer brain-infiltrating neutrophils, and a more favorable immune environment that reduced hemorrhagic transformation compared to that observed in the isochronic controls [15]. This highlights that bone marrow can manipulate peripheral blood immune cells to counteract the negative effects induced by stroke. These immune-driven effects may have broad implications for both the brain and systemic aging. Blood replacement therapy improves outcomes in post-stroke mice by reducing neutrophils in the blood and MMP-9 levels in the brain and blood after stroke, decreasing infarct size and cell death, and lowering levels of post-stroke proinflammatory cytokines (IL-6, IFN-γ, and TNF-α) and chemokines (CXCL1 and CXCL2). This treatment may provide a mechanism for alleviating post-stroke injury in aging mice [15]. Splenectomy, which removes peripheral immune cells, reduces stroke-induced inflammation and injury in aged mice, thereby improving cognitive recovery [101]. Although the role of NETs is to trap pathogens and help fight infections, NETs in the plasma trap other blood cells to form pathological thrombi. Furthermore, NETs increase inflammatory cytokine levels and trigger proinflammatory microglial subtypes [102]. For example, Denorme et al. demonstrated that elevated plasma NETs in patients with ischemic stroke were associated with worse stroke outcomes and administration of neonatal NET-inhibitory factors improved stroke outcomes in older mice [103].

Neutrophil adhesion to endothelial cells may lead to a cumulative stalling and prevention of erythrocyte movement through the microvascular system, thus leading to infarct enlargement [104]. Treatment with the neutrophil-depleting antibody anti-Ly6C significantly improved long-term neurological deficits and behavioral recovery after ischemic stroke in aged mice compared with young mice [97]. Roy-O'Reilly et al. showed that the response of neutrophils to ischemic stroke had a unique age-dependent pathogenicity that may be related to elevated levels of potential mediators of neutrophil activation (IL-6, IL-8, and CXCL1) [97]. Higher neutrophil counts in middle-aged and older populations are associated with an increased risk of fatal stroke [96]. This suggests that the increased priming of neutrophils in elderly individuals or mice may occur secondary to inflammaging, which promotes neutrophil activation via elevated inflammatory factors.

These results suggest that neutrophil pathogenicity in ischemic stroke is substantially correlated with aging and that neutrophil-targeted therapies may benefit older patients more significantly than younger patients. However, the clinical translation of these results presents several challenges. Therefore, the associated mechanisms and potential treatments must be evaluated in older animals to better translate the results of preclinical studies.

#### 4.2.2 Monocytes/macrophages

Mononuclear phagocytes, including microglia, macrophages, and monocytes, migrate to the site of ischemic stroke lesions and coordinate immune responses. Aging has not been demonstrated to significantly affect the total number and frequency of monocytes in humans; however, it causes substantial changes in the relative distribution and function of mononuclear phagocytes.

Monocytes in mice can be categorized into the following two subsets: inflammatory (Ly6C^hi^CX3CR1^int^ CCR2+) and patrolling (Ly6C^low^CX3CR1^hi^CCR2-) monocytes. Inflammatory and patrolling monocytes are functionally equivalent to CD14^hi^CD16^low^ and CD14^low^ CD16^hi^ phenotypes in humans, respectively [105]. These cells are recruited from the bloodstream after ischemic brain injury. For example, inflammatory CCR2+ monocytes typically leave the bone marrow or spleen to enter the bloodstream and infiltrate inflamed tissues in a CCR2-dependent manner, which mediates the recruitment of MDMs. The spleen is a source of MDMs in the brain and represents an essential factor in modulating immune responses after stroke [106]. Splenectomy restored peripheral inflammatory cell levels and reduced the infiltration of peripheral immune cells into the brain in aging stroke mice, and splenectomy after reperfusion also improved neurobehavioral and infarct outcomes [101].

Studies have demonstrated that inflammatory CCR2+ monocytes benefit older brains after stroke by promoting phagocytic activity, such as clearing debris [14, 16], promoting M2 macrophage polarization, and preventing hemorrhagic transformation [107]. However, persistent activation can lead to chronic inflammation [16]. Autologous transplantation of M2-like MDMs into the CSF can promote motor and cognitive function recovery after stroke without affecting the infarct volume [41], and it may represent a new strategy to promote the recovery of patients who experienced stroke [41]. Microglia modulate functional outcomes of ischemic stroke through neurogenesis, angiogenesis, and neuroplasticity [108]. Similarly, monocytes and macrophages (MMs) also regulate neural function in this manner. Repair of CCR2 monocytes contributes to angiogenesis, tissue remodeling, and functional recovery after acute ischemic stroke [16]. The early elimination of circulating monocytes using the anti-CCR antibody MC-21 enhances striatal neurogenesis [109]. Thus, elucidating the cellular mechanisms of MM in functional recovery from ischemic stroke and determining the underlying molecular mechanisms may lead to new therapeutic strategies for aging-related diseases.

#### 4.2.3 Natural killer (NK) cells

NK cells are innately toxic lymphocytes that cause multiple types of brain damage [63]. NK cells enhance local inflammation, thus leading to cellular necrosis through IFN-γ expression; mediate cytolysis of ischemic neurons via perforin; and disrupt the BBB in response to IFN-inducible protein 10. Moreover, CX3CR1 expression in NK cells is required for neutrophil recruitment, and the presence of CX3CR1 leads to increased CX3CL1 production by ischemic neurons, which attract NK cells to the site of brain injury [110]. Zhang et al. hypothesized that NK cells are involved in cerebral ischemia via IFN-γ and promote neuronal necrosis [111]. After ischemia, local inflammation and neuronal hyperactivity rapidly increase because brain infarction worsens with the loss of NK cell tolerance [110]. Although NK lymphocytes cause ischemic injury, Tregs present in ischemic tissues protect against inflammation-induced neurological damage via IL-10.

Furthermore, the NK cell response is compromised in elderly individuals. A decline is observed in the NK-cell subset CD56^bright^, which has a strong capacity for cytokine and chemokine production, including IFN-γ and TNF-α, and an increase occurs in the NK-cell subset CD56^dim^, which has cytotoxic potential [112]. NK cells become hyperactive with age and play a role in inflammation through changes in TLR function and a shift in subtypes. The number of NK cells was significantly increased in the postmortem brain tissue of elderly patients and aged mice. Notably, with aging, the number of NK cells increases while the number of cytokines and chemokines released by NK cells; the efficacy of NK cell cytotoxicity; degranulation, expression, and release of perforin; and length of telomeres (TL) of the NK cell subpopulation decrease [18]. In addition, neuroendocrine signaling also influences NK cell senescence.

Although differences in the effects of aging and stroke on the NK-cell phenotype have been elaborated, most studies have only considered age-related changes in brain NK cells. Deciphering intercellular interactions and communication between the brain and peripheral systems and investigating age-related heterogeneous changes in peripheral circulating NK cell populations have important implications for controlling NK cell activity and identifying therapies to target aging and age-related diseases.

#### 4.2.4 Dendritic cells

DCs are the most effective antigen-presenting cells (APCs) and represent the primary link between innate and adaptive immunity, and the two major DC subgroups are myeloid DCs (mDCs), which are also known as conventional DCs (cDCs), and plasmacytoid DCs [113]. Aged DCs exhibit impaired PI3K signaling and increased autoantigen (DNA) reactivity, leading to increased inflammation and reduced antigen migration and uptake. Reduced PI3K acts as a negative regulator and is linked to age-related decreases in DC migration and TLR signaling [20]. The treatment of young mDCs with PI3K inhibitors supports the role of PI3K pathways in the impaired ability of aged mDCs to inhibit LPS-induced Akt phosphorylation but increase p38 phosphorylation, which is associated with increased cytokine production, reduced antigen uptake, and decreased migration [114]. The overall result of reduced PI3K activation is higher activation of the NF-kB pathway, which further contributes to inflammaging by producing proinflammatory cytokines (e.g., TNF-α) [20]. Thus, further investigations are needed to directly assess the causal role of DCs in brain aging.

Recent evidence suggests that PI3K/Akt pathway activation protects rat brains from cerebral stroke, promotes angiogenesis for brain repair [115], attenuates neuroinflammation and neuronal apoptosis after cerebral hemorrhage in mice [116], and exerts neuroprotective effects against cerebral ischemia-reperfusion (IR) injury in rats [117]. Therefore, further studies should focus on the role of the PI3K/Akt pathway in the effects of aging on stroke. DCs are detected within days of stroke onset, and they accumulate at the core of the ischemic infarct. The secretion of IL-23 by cDC2s and induction of IL-17 in γδ T cells promote neutrophil infiltration of the ischemic brain, whereas the interruption of the IL-23/IL-17 cascade improves neurological outcomes after stroke [118]. Li et al. used a scRNA-seq system to analyze the immune population in the post-stroke aged brain and found that IL-1β was primarily expressed in DCs, neutrophils, and macrophages. Additionally, they observed a significant increase in DCs, particularly DC1 cells, in the post-stroke aged brain. Studies have also reported that TBI affects the differentiation and distribution patterns of DCs and reduces ROS levels [119]. Therefore, a more precise understanding of the molecular mechanisms that lead to DC deficiency after TBI is required to effectively treat infections in patients who experienced stroke.

Overall, these findings suggest that immunosenescence-related innate immune cell dysfunction promotes the activation of brain-resident immune cells and the infiltration of peripheral immune cells through increased cytokine and chemokine production and adhesion molecule expression, which play pivotal roles in the pathogenesis of stroke in elderly individuals.

#### 4.2.5 T cells

The thymus is crucial for the maturation and development of diverse T-cell populations. T cells can be classified into subsets, including Th, cytotoxic T (Tc), and Treg cells, based on their various immune response roles [120]. Thymic degeneration occurs with aging and is believed to be correlated with changes in sex hormone levels and IL-7, a hematopoietic growth factor generated by stromal cells in the thymus and bone marrow [121]. Due to lifelong exposure to various pathogens and antigens, the number of naïve cells is reduced while the population of memory cells is increased [121]. Aged naïve CD4+ T cells are less responsive to antigens and APCs, thus preventing them from expanding, producing cytokines, and differentiating, which is similar to the findings for young mouse CD4+ T cells [122]. The most striking feature of senescent CD8+ T cells is a decrease in the T-cell receptor (TCR) repertoire, which is harmful because a diverse TCR is essential for protection against viral infections. The main features of these senescent T cells are their low proliferative potential upon activation, shortened telomeres, low telomerase activity, excessive ROS production, and constitutive p38 MAP kinase activation, and they prevent TCR-based signaling. In addition to supporting the definition of inflammaging, these findings can be extended to explain age-related thymic atrophy, memory conversion, TCR pool shrinkage, and increased migration to non-lymphoid tissues.

Th cells that express the CD4 surface marker assist other lymphocytes or secrete cytokines to regulate immune system activity. The most common Th cells are Th1, Th2, and Th17. The impaired capacity of CD4+ T cells to differentiate into functional subsets results in numerous dysregulated responses and represents one factor that may favor inflammaging. Specifically, decreased cognate assistance to B cells results in diminished humoral immunity, and an increased ratio of Th17 to Treg cells promotes basal proinflammatory conditions [123]. For example, Dolati et al. found that an increase in the peripheral Th17/Treg ratio in elderly patients with ischemic stroke contributes to stroke pathogenesis [124]. Peripheral Th17 levels are elevated in patients with acute stroke, which leads to the upregulated expression and function of proinflammatory factors [125]. A previous study performed a 1-year follow-up of 106 elderly patients after stroke and showed that CD4+CD28- T cell counts in the peripheral blood are associated with an increased risk of recurrent stroke and death, suggesting that this subpopulation of T lymphocytes has proinflammatory and tissue-damaging potential [126].

Cytotoxic T cells that express the CD8 surface marker (CD8+ cytotoxic T cells) cause neuronal death and exacerbate brain damage through cell interactions and granzyme and perforin secretion [21]. The aging stroke brain increases the release of CD8+ T cell chemokines, signaling of proinflammatory factors, expression of adhesion molecules, and number of CD8+ T cells extravasating into the perivascular and parenchymal regions, which promote acute ischemic injury [127]. Additionally, a significant increase in CD8+ T cells was observed in the meninges, parenchyma, and CP of aged mice. Selvaraj et al. confirmed that the delayed egress of CD8 + T cells into the ischemic brain negatively affects functional recovery in tMCAO mice [128]. Astrocyte-derived IL-15 enhances the responses of CD8+ T and NK cells, increases brain inflammation, and exacerbates ischemic brain injury [84]. Interestingly, a recent study found that *in vivo* CXCL13 neutralization or CD8+ T cell depletion restores sensory neuron axonal regrowth, thereby promoting functional recovery [129]. They proposed an effective clinical method for neurological repair in elderly individuals that consisted of antibody-mediated regulation of neuron-immune cell crosstalk, and it has the potential to improve immunosenescence-related diseases (e.g., stroke).

However, not all T-cell subtypes deteriorate with age; for example, the quantity and functionality of mouse and human Tregs increase. Increasing evidence suggests that Treg accumulation is amplified in young mice after ischemic stroke through IL-2, IL-33, and TCR recognition, and it enhances neurological recovery during the chronic phase of ischemic brain injury [130]. Brain Tregs inhibit neurotoxic astrogliosis by producing amphiregulin, which reduces neuroinflammation and neuronal damage and promotes neurological recovery after stroke [130]. Additionally, Tregs significantly improve long-term function after stroke by enhancing the repair activity of microglia and promoting oligodendrocyte regeneration and damaged white matter repair [131]. Although Tregs have been found to protect against neurovascular destruction after stroke by inhibiting peripheral neutrophil-derived MMP-9, these findings were based on a study conducted in young animals [132]. Nonetheless, the association between Tregs and age-related brain improvements in stroke is of great research value. In contrast, Kleinschnitz et al. confirmed that Treg depletion in DEREG and Rag1 mouse models significantly reduced infarct size and improved neurological function after stroke [133]. Similarly, Treg expression increases thrombosis in the brain microvasculature, thereby promoting infarct size and worsening functional outcomes [134]. We hypothesize that poorer prognoses in older patients who experienced stroke may be associated with increased Treg numbers. However, studies on the relationship between Tregs and stroke in elderly patients are limited. Therefore, whether Tregs are beneficial or detrimental to stroke outcomes remains controversial and warrants further investigation.

#### 4.2.6 B cells

Similar to the findings for T cells, aging alterations in B cells involve a decrease and an increase in naïve and memory B cells, respectively. As part of the humoral immune system, B cells play a role in immune activation and resistance to infection. The presence of B cells in the brain is significantly increased in disease/injury states that cause damage to the BBB integrity [135], suggesting an essential and potentially protective role for B cells in the context of stroke. Recent studies by Ortega et al. indicated that the protective role of B cells after stroke might be associated with their capacity to migrate to various ipsilateral and contralateral sites of injury within the brain parenchyma, promote neuronal viability, maintain dendritic complexity, stimulate neurogenesis through IL-10-dependent mechanisms, and contribute to long-term motor function recovery [136]. However, B cells exposed to prolonged inflammation are likely to produce pathogenic antibodies and proinflammatory cytokines. With age, beneficial antibody production by B cells is impaired and the subsets are altered. Age-related B cells (ABCs) are a proinflammatory B-cell subset that secretes substantially high levels of TNF-α and inhibits the differentiation of normal B cells, and they are more prevalent in the bone marrow of aged mice [137]. Several ABC biomarkers are associated with decreased class-switch recombination and somatic hypermutation of immunoglobulin genes [23]. In addition to the age-related decline in high-affinity protective antibodies, protective immunity does not last long after vaccination [23]. These shifts increase the risk of infection among elderly individuals. Studies have demonstrated that acute infections increase the risk of stroke while active infections during stroke dramatically decrease BBB integrity, infarct volume, and functional outcomes [24].

CD11b^high^ B cells can increase the microglia phenotype via regulatory cytokines (TNF-α) in immune-dysregulated aged stroke mice [138]. Additionally, one study found that the total number of B cells is significantly increased in the dura mater of aged mice [139]. In this study, differentially expressed genes between ABCs and mature B cells were identified using scRNA-seq sequencing, and the most upregulated genes were Apoe, Ly6a, Ighm, Igkc, and Cd2. These results suggest that during aging, ABCs and plasma cells infiltrate the mouse meninges via circulation [139]. This also confirms the existence of a direct vascular channel between the skull and dura mater that can transport immune cells. Interestingly, studies have shown that ABC dysfunction occurred after 60 years of age while no dysregulation was observed in genome-wide expression profiles of B cells from donors under 60 years of age [140]. Clinical and experimental differences emphasize the complexity of "aging," which varies in animals, individuals, and different cells of individuals. Thus, future investigations should combine individuals and animals of different ages with different genotypes, comorbidities, and environmental exposures using multidimensional and longitudinal immune mapping after ischemic stroke.

Human studies [141] have evaluated partial cellular infiltration in areas of liquefied necrosis (i.e., weeks to months) in aged stroke brains and found that CD68+ macrophages, T cells (CD4+ and CD8+ cells), and B cells (CD20+ cells) were all present at the infarct site. Compared with the acute phase, which showed elevated levels of 25 cytokines and 19 chemokines, the liquefied necrotic phase showed significant reductions in most inflammatory factors. Despite substantial abatement of the inflammatory response from the acute to the liquefactive necrotic phase, proinflammatory factors (IL-6 and monocyte chemoattractant protein-1 [MCP-1]) remained chronically elevated at low levels. Moreover, in the peripheral blood of elderly individuals, late memory or exhausted B cells [double-negative (DN) B cells] increase, which is similar to that observed for ABCs [142]. DN B cells express SASP markers and activate NF-κB, which leads to increased production of proinflammatory cytokines (IL-6, IL-8, and TNFα) and inflammatory microRNAs (miR155/16/93), and they are reliant on metabolic signaling through MAPK [143]. Ruschil et al. studied the involvement of DN B cells in various active neuroinflammatory diseases and vaccinations and found that they play a role in protective and pathogenic immune responses [144]. The telomeres of aged donor DN B cells are very short compared to those in young donors [145]. However, little is known about the formation and function of DN B cells and their involvement in other CNS diseases, including stroke; thus, additional studies are required on the pathological and protective antigen-targeted immune response of DN B cells in stroke.

These studies have provided important information on age-related factors that may be associated with poorer outcomes and elevated risk of infection after stroke and show that these factors are possible targets for lowering the influence of age on stroke. Future work is also needed to establish a chronological sequence of immune cell responses during aging in stroke patients. This chronology could provide a comprehensive analytical framework to identify immune events associated with adverse clinical outcomes after stroke.

## 5. Molecular Regulators of Cellular Senescence in Stroke

**Figure 2.**
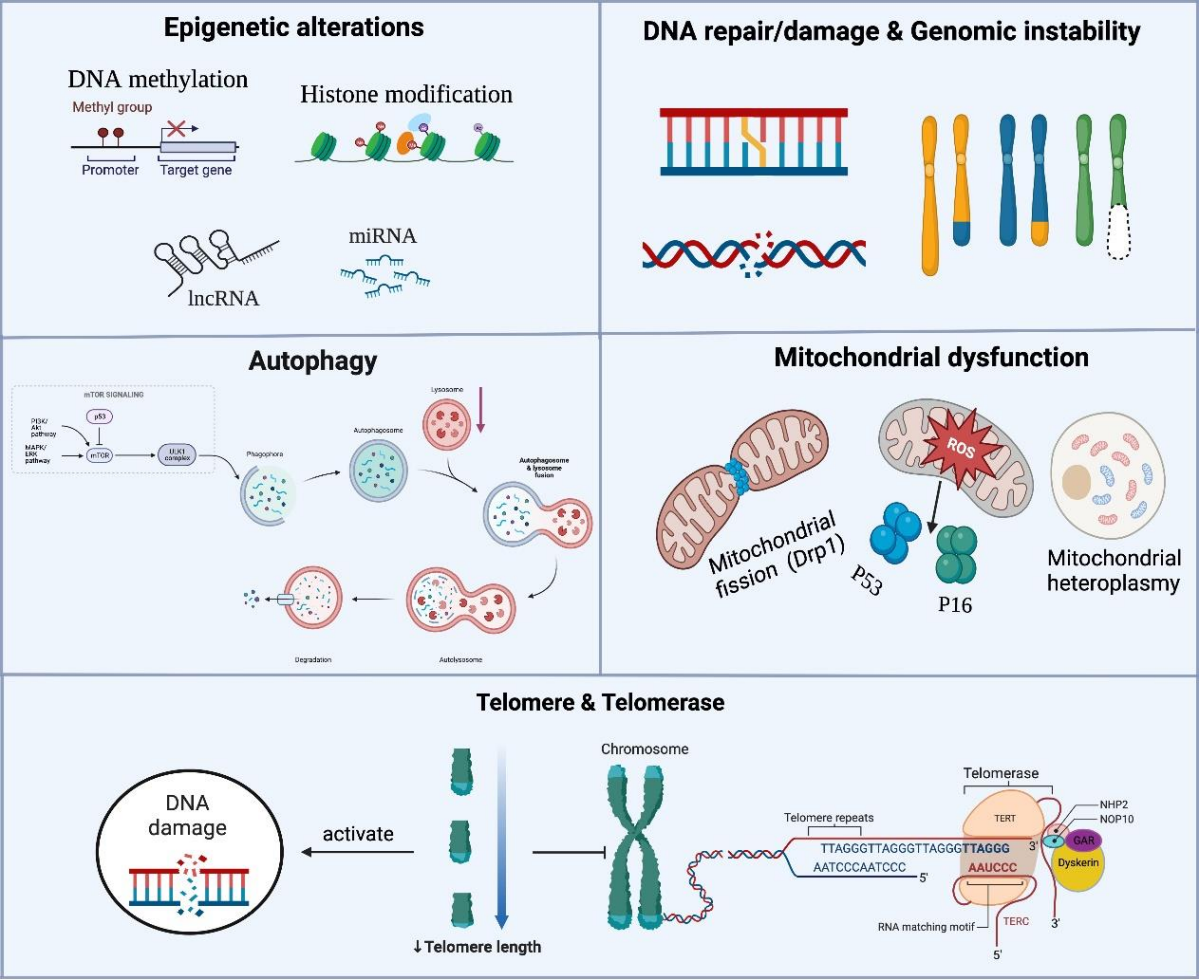
**Molecular regulators of cellular senescence**. Mechanisms that drive cellular senescence include telomere attrition, DNA repair and genomic instability, mitochondrial dysfunction, epigenetic alterations, and impaired autophagy.

In this section, we discuss the molecular mechanisms that drive aging under pathological conditions, including telomere attrition, DNA repair, genomic instability, mitochondrial dysfunction, epigenetic alterations, and impaired autophagy. Although their relevance to stroke has been previously discussed, their causal relationship with age-dependent stroke impairment requires further investigation (Fig. 2).

### 5.1 Telomere attrition

Telomeres are pleiotropic and involved in chromosomal homeostasis, gene expression regulation, and stress-related signaling pathway modification [146]. Substantial evidence supports the relationship between TL shortening and an increased incidence of age-related disorders in humans, such as ischemic stroke [147], atherosclerosis [148], and cardiovascular disease [149]. Therefore, TL may be a risk factor for stroke development and progression in older individuals. Additionally, we discovered that telomere shortening activates the DNA damage response (DDR), which leads to cell senescence or death, thus indicating a strong relationship between DDR and telomere maintenance. Telomere attrition leads to p53-dependent sirtuin inhibition, which triggers metabolic remodeling, and triggers the DDR via poly(ADP-ribose) polymerase and ataxia telangiectasia-mutated, which reduces genomic stability, ultimately leading to cellular senescence [150].

Yetim et al. found that a shorter TL and more severe clinical phenotype indicated lower resistance of the cerebral tissue to ischemia [151]. Zhang et al. analyzed the association between TL and recurrent stroke and found that the cumulative rates of stroke recurrence were negatively correlated with TL, thus highlighting the substantial link between low TL and stroke recurrence in individuals older than 65 years of age [147]. However, they did not find significant interactions among TL, age, and stroke recurrence when comparing younger and older participants [147]. Similarly, Cao et al. applied a two-sample Mendelian randomization approach and did not find strong evidence to support shorter TL having an effect on ischemic stroke [152]. In a subsequent study, these authors demonstrated that the relationship between TL and ischemic stroke risk was nonlinear [153]. Therefore, further research is required to clarify the potential correlation between novel technical approaches and design protocols.

Telomerase is a TERC-coupled reverse transcriptase involved in maintaining telomeres, stabilizing the genome, regenerating tissues, and safeguarding mitochondria [154]. Mojiri et al. previously demonstrated that the transient introduction of a modified mRNA encoding human telomerase reverse transcriptase (hTERT) into senescent human cells can lengthen telomeres, reverse aging-related traits, and restore replicative ability [155]. Recently, these authors also showed that hTERT therapy lengthens telomeres, improves endothelial gene expression and function, increases sirtuin 1 (SIRT1) expression, lowers DNA damage markers, and attenuates inflammatory cytokine release [156]. TERT deficiency exacerbates BBB dysfunction, inflammatory responses, and oxidative stress in stroke mice, leading to neurological deficits and increased infarct volumes [157]. In young cerebral ischemic rats, TERT expression promotes postinjury angiogenesis and improves BBB integrity [157]. Moreover, TERT exerts beneficial effects during ischemia via the Notch-1 signaling pathway [157]. We hypothesize that TERT positively affects aged animals with stroke. Moreover, the potential age-regulated role of TERT may have therapeutic implications on the risk of ischemic stroke and functional outcomes in the elderly population.

### 5.2 DNA repair and genomic instability

Although senescent cells develop naturally with age, the presence of persistent cellular stress accelerates aging [158]. Cellular senescence can be effectively triggered by double-strand break aggregation during DNA damage [159]. Physiological alterations caused by DNA damage may increase genomic instability, and the decline in homeostasis may be exacerbated with aging. Time-related accumulation of DNA damage is partly due to endogenous factors, including ROS and advanced glycation end products, and partly due to exogenous factors, including ultraviolet light and chemical compounds in food and water [160]. These effects gradually impair cellular function and increase the risk of age-related chronic diseases.

Notably, ineffective DNA repair is a critical contributor to premature aging and neurodegenerative disorders [161]. Inflammatory reactions have also been observed in progeroid mice with deficient DNA repair mechanisms [162]. Interestingly, calorie restriction significantly slows premature aging in DNA repair mutant mice, which is likely related to the reduced levels of ROS and other reactive chemicals because such changes also reduce DNA damage [163]. The *Caenorhabditis elegans* model for defective transcription-coupled repair in Cockayne syndrome accelerates neurodegeneration, thus revealing the driving factors of DNA damage in the pathogenesis of age-related neuronal diseases [164]. After ischemic brain damage, enhanced DNA repair ability may boost angiogenesis and neurovascular unit remodeling [165]; however, further research is required to validate this hypothesis.

Overall, therapies designed to address the root causes of aging-related multimorbidity (e.g., ischemic stroke) should focus on restoring genomic integrity by reducing DNA damage and enhancing DNA repair.

### 5.3 Mitochondrial dysfunction

Mitochondrial dysfunction, which is a cause and consequence of cellular senescence, is defined as a decline in the respiratory capacity of each mitochondrion and a reduction in the mitochondrial membrane potential at a steady state, and it is typically accompanied by an increase in the formation of oxygen-free radicals [166]. Mechanisms contributing to mitochondrial dysfunction during aging and senescence include genomic instability (mitochondrial DNA mutations), calcium overload, dysregulated nutrient-sensing pathways, imbalanced NAD+/NADH ratios, and impaired mechanisms involved in mitochondrial mass homeostasis [167].

After ischemia, mitochondrial dysfunction initiates a cascade of events that leads to neuronal death. Specifically, mitochondrial depolarization caused by cerebral ischemia results in decreased adenosine triphosphate synthesis, excessive ROS production, and increased PINK1 accumulation [168]. The pathophysiology of cerebral ischemia is significantly influenced by excessive ROS levels, which affect the brain tissue structure and function [168]. Studies have demonstrated that dynamin-related protein 1 (Drp1) plays a crucial role in ischemic stroke and infarct volume decreases when Drp1 is downregulated [169]. Additionally, after Drp1 knockdown, oxidative stress and mitochondrial ROS production decreased [170]. Therefore, reducing the negative consequences of oxidative stress by identifying the mechanisms underlying apoptotic and necrotic neuronal damage and aging is a promising strategy for treating ROS-related disorders, such as ischemic stroke.

Mitochondria are primary regulators of NLR family pyrroline-containing structural protein 3 (NLRP3) inflammasome activation and cell death [171]. Moreover, the NLRP3 inflammasome has been reported to mediate inflammatory reactions during ischemic stroke and ROS may be required for NLRP3 inflammasome activation [172]. Pro-caspase-1 is transformed into caspase-1 upon activation of the NLRP3 inflammasome, and caspase-1 cleaves pro-IL-1β and pro-IL-18 into their active proinflammatory cytokines. Subsequently, these cytokines are released into the extracellular environment, which triggers autophagy and pyroptosis [173].

Peroxisome proliferator-activated receptor γ coactivator 1α (PGC-1α) is one of the primary mediators of mitochondrial function, and its expression and activity have been linked to age-related neurodegenerative disorders, such as ischemic stroke [173]. PGC-1α also affects energy generation and ROS defense mechanisms. However, the expression and activity of PGC-1α decrease with age [174]. Notably, recent studies have shown that the activation of the PGC-1α signaling pathway may provide a protective mechanism against ischemic brain injury by suppressing neuroinflammation [12, 175]. Moreover, PGC-1α activation ameliorated mitochondrial biosynthesis and prolonged lifespans [176]. Thus, the mechanisms underlying PGC-1α regulation in aging animals after ischemic stroke and its impact on functional recovery will likely become a focus of future research on the aging brain.

### 5.4 Epigenetic alterations

Epigenetic alterations can be influenced by environmental factors, including aging, stress, and diet, which regulate physiological status [177]. With aging, epigenomic regulation has become increasingly important for differentiating immune cell phenotypes and controlling inflammatory responses to internal and external stress. DNA methylation, histone modification, and RNA-based mechanisms are the primary epigenetic mechanisms [178]. Increasing evidence shows that epigenetic mechanisms modulate cerebrovascular pathophysiology in humans and experimental stroke models [178].

Studies have reported that in the presence of DNA damage, the loss of H3K27Me3 induces cellular senescence by activating p16 and p21; therefore, its loss or decrease is considered a hallmark of senescent cells [179]. Moreover, Schwab et al. examined the brains of patients with mild TBI and found that they showed a loss of Lamin B1 and H3K27Me3 expression in glial cells, which is consistent with reports on senescence [179, 180]. However, the role of H3K27Me3 in cerebrovascular diseases is controversial. Wang et al. found that H3K27Me3 was significantly upregulated in rats with ischemic stroke and the administration of GSK-126 to rat brains decreased the level of H3K27me3 and reduced neuronal apoptosis [181]. Similarly, stroke is a disease of aging, and studies in Drosophila have demonstrated that excess H3K27me3 levels increase with age [182]. Therefore, additional research must be performed to fully understand the dynamics of DNA damage repair, genome maintenance, and H3K27me3 in senescent cells and their impact on ischemic stroke.

DNA methylation affects gene expression and chromatin organization, and it is the biomarker of aging that has received the most attention. Changes in methylation have been associated with complex age-related diseases, such as stroke. In 2014, Soriano-Tárraga et al. reported global hypomethylation in ischemic stroke cases compared to controls and age-related DNA hypomethylation using LUMA analysis [183]. Subsequently, in 2016, they first found that the epigenetic age of patients with ischemic stroke was 2.5 years older on average than that of the controls, and they concluded that it is possible to predict chronological age by measuring age-related changes in DNA methylation at multiple cytosine-phosphate-guanine sites across the genome (called biological age) [184]. Biological age is a potential biomarker of stroke risk. In 2017, the effects of analyzing biological age on prognosis in patients 3 months after ischemic stroke further provided accurate measurements of these effects on individuals with stroke who affect aging and potential longevity [185]. Approximately 500,000 DNA methylation sites in individuals with ischemic stroke treated with antiplatelet medications were examined by Gallego-Fabrega et al., who discovered a link between elevated stroke recurrence and hypomethylation of the "inflammation control" genes protein phosphatase 1A and TNF receptor-associated factor 3 [186, 187].

Histone methylation influences stroke severity with age in animals and patients who experienced stroke [188]. Additionally, investigations have suggested a relationship between histone methylation and proinflammatory cytokines [189]. Inflammation is a potential cause of ischemic stroke, and DNA methylation and histone acetylation alter the promoter region of TNF-α [190]. Studies have shown that the TNF-α promoter region becomes methylated with age and correlates with decreased mRNA expression in animal spleens [191]. Similarly, the NF-κB family stimulates the expression of cytokines and genes related to apoptosis and senescence [192], and NF-κB levels can be modulated by epigenetic mechanisms, such as the acetylation of histone H3 by the H3 lysine four methyltransferase SET7/9 [193]. Additionally, Liu et al. observed a significant increase in the expression of the histone demethylase lysine-specific demethylase 4A (KDM4A) and the proinflammatory cytokines IL-1β and TNF-α in rats with MCAO and found that KDM4A inhibition improved functional recovery from ischemic stroke by suppressing NF-κB activation and subsequent neuroinflammation [194].

Therefore, the effect of epigenetic changes on the aging process and the risk of stroke must be further studied. Notably, these chronological or biological age-based epigenetic clocks can predict the severity of ischemic stroke and consequent exacerbation of chronic diseases.

### 5.5 Impaired autophagy

Autophagy is a crucial intracellular degradation process that contributes to the development of various neurodegenerative disorders by eliminating damaged or dysfunctional proteins and organelles [195]. Additionally, the onset and progression of the inflammatory process appear to be closely associated with the autophagy pathway. Specifically, aging decreases autophagy and other processes that control proteostasis, such as proteasome activity [196]. This results in the accumulation of misfolded protein aggregates, which trigger inflammatory pathways. Notably, the geroprotective effect of the immunosuppressant rapamycin responds to cellular damage by reducing mTORC1 signaling, which increases with age [197]. Several autophagy genes, including BECN-1, ATG-5, and ATG-7, are downregulated in the brains of older patients, and the brains of aged rodents show increased mTORC1 activity and decreased ATG protein levels, suggesting that aging is associated with reduced autophagic function [198].

However, a consensus has not been reached regarding the protective or detrimental role of autophagy since both positive [199] and negative [200] regulation of autophagy have been found to promote neuroprotection in *in vivo* models of cerebral ischemia. Although studies have demonstrated that autophagy protects neurons, glia, and endothelial cells from ischemic injury and improves clinical outcomes [201], other studies have indicated that excessive autophagy may damage brain cells [202]. Despite these contradictory results, it is generally accepted that moderate autophagy protects against ischemia while excessive autophagy may cause cell death. Therefore, the severity of ischemic stroke and the poor outcomes in older individuals may be influenced by impaired autophagy, which is a hallmark of aging.

## 6. Biomarkers of Cellular Senescence in Stroke

Strong evidence exists that brain cell senescence influences the pathogenesis of neurodegeneration [203]. However, the possibility that senescent cells are involved in the causative mechanism of stroke-induced brain injury has recently been explored. Therefore, biomarkers of multifactorial processes must be identified to accurately assess cellular senescence linked to stroke.

### 6.1 Senescence-associated secretory phenotype

Since the SASP is significantly activated in most senescent cells, we present the implications of the SASP in ischemic stroke. Senescent cells release various substances known collectively as the SASP, and they include MMPs (e.g., MMP-1, -3, -10, -12, and -13), growth modulators (e.g., epiregulin, epidermal growth factor, and hepatocyte growth factor), angiogenic factors, proinflammatory cytokines (e.g., IL-1β, IL-6, IFN-γ, and GM-CSE), and chemokines (e.g., IL-8, GRO-a, MCP-2, and MCP-4) [204]. The SASP promotes and spreads senescence in an autocrine or paracrine manner as a trait of senescent cells. Senescent cells develop many diseases by secreting SASP factors that transmit aging to neighboring cells, thus leading to inflammaging.

Baixauli-Martín et al. showed that the ischemic hemisphere in rats with ischemic stroke had considerably greater levels of the SASP cytokines IL-6, TNF-α, and IL-1β than the nonischemic hemisphere, particularly in the subcortical (striatal) region [205]. NO induces cellular senescence and regulates the expression of SASP factors by modulating many signaling pathways (e.g., NF-κB and STAT3) [206]. Furthermore, Lim et al. indicated that nitric oxide synthase 2 expression in the immunostaining of ischemic stroke regions significantly increases during IR injury in the brain [207]. A recent study showed that gene expression of proinflammatory cytokines (IL-6, TNF-α, CXCL1, and Cxcr2) in the brains of tMCAO mice was restricted to the anatomical infarct region 72 h after ischemic injury [208], and the findings are consistent with the transcriptional profile of the SASP. This study identified increased cellular senescence markers in the penumbra during the first 30 min after ischemic stroke and higher cellular senescence markers in the ischemic core over time [208]. These changes were related to the development of stroke. Indeed, SASP mediators play crucial roles in the onset of senescence and support the notion that neuroinflammation and senescence are related to stroke neuropathology.

### 6.2 Senescence associated-β-galactosidase activity or lipofuscin accumulation

Senescence-associated-β-galactosidase (SA-β-gal), which is defined as the β-galactosidase activity detectable at pH 6.0 in senescent cells, is the most frequently used biomarker of aging and senescent cells [209]. SA-β-gal activity cannot be detected in quiescent or differentiated cells, and it is closely correlated with senescent cells [209]. Lipofuscin, which is a biological marker of aging, is primarily responsible for the increase in lysosome size and volume in senescent cells [210].

Lim et al. showed that SA-β-gal staining *in vitro* following oxygen-glucose deprivation/reoxygenation injury induced senescence in rat cortical astrocytes but not in the tMCAO rat model [207]. A recent study has shown that the levels of SA-β-gal-positive cell staining gradually increased from 1 to 14 days and peaked at 7 days after injury in the TBI mouse model [206]. However, in a tMCAO mouse model, Torres-Querol et al. found no SA-β-gal-positive cell expression in the infarcted and contralateral brain regions [208]. This could explain the shorter post-ischemic time window (3 days after ischemic stroke) [208]. Many researchers have used lipofuscin rather than the SA-b-gal assay to identify senescent cells [205] and suggested that the Sudan black B (SBB) assay for lipofuscin overcomes the main drawback associated with the use of the commonly accepted senescence marker SA-b-gal. In the first 24 h after ischemia/reperfusion, SBB-positive lipofuscin granules were dispersed, and their density increased with time; moreover, these granules appeared in aggregates in infarcted brain tissue [205]. Thus, developing a more efficient and accurate commercial kit represents a promising method of detecting SA-β-gal activity.

### 6.3 Cell-cycle arrest markers (p21, p53, and p16)

**Figure 3.**
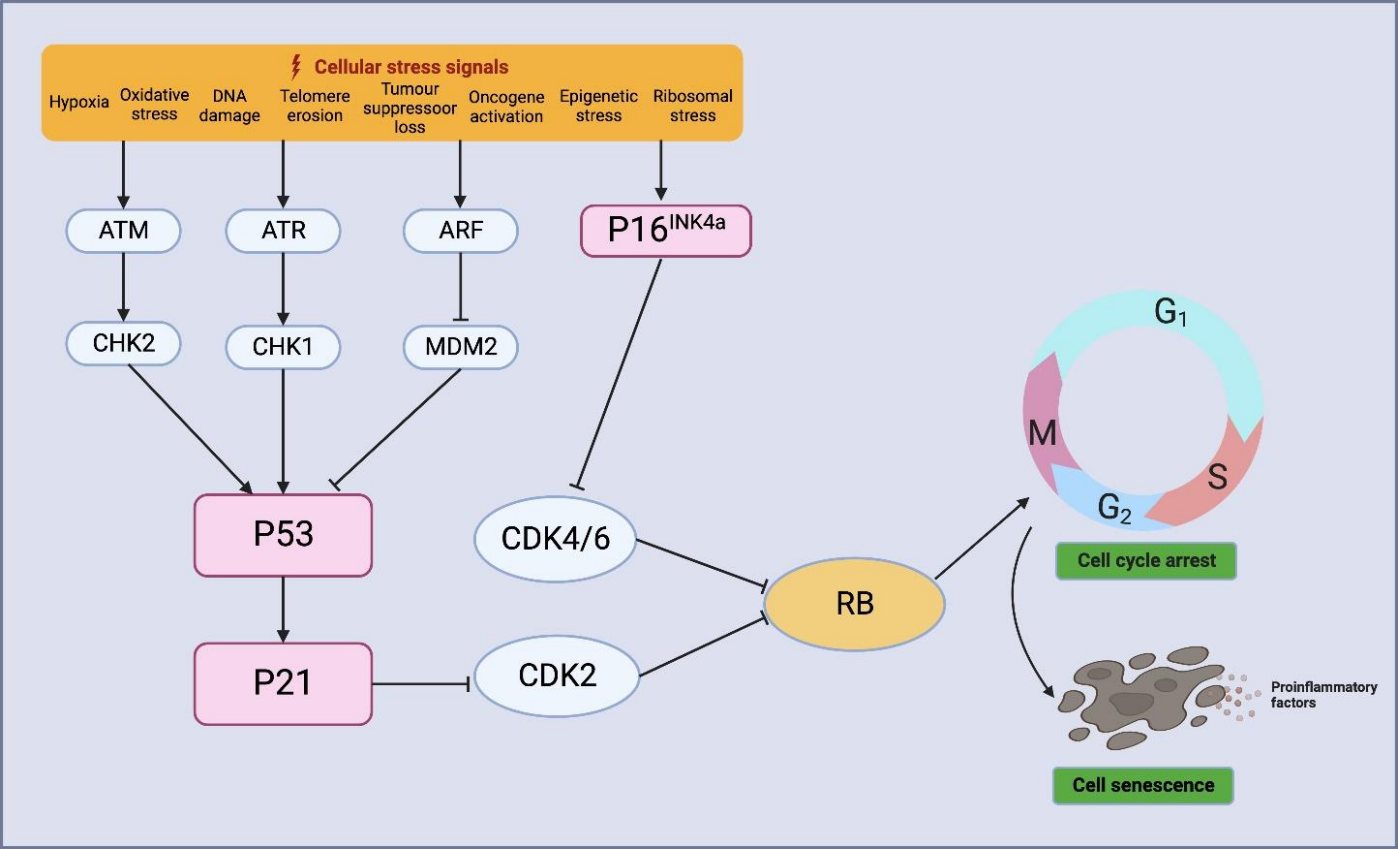
**The pathways that regulate cell cycle arrest**. Various cell-intrinsic and -extrinsic stressors can activate the cellular senescence process. These stressors involve multiple cellular signaling cascades, eventually activating p53 and p16 INK4a. The kinase cascades involving the apical kinases ataxia telangiectasia mutated and ATR and the downstream kinases CHK2 and CHK1 activate p53 and promote p21 activation, which induces temporary cell cycle arrest by inhibiting the cell cycle protein CDK2. p16 INK4a suppresses cell cycle progression by targeting the CDK4 and CDK6 complexes. Both p21 and p16 INK4a function by inhibiting RB inactivation, which maintains the repression of E2F target genes needed for the onset of the S-phase. RB, retinoblastoma.

The primary pathways that regulate permanent cell cycle arrest are p53/p21^CIP1^ and p16 INK4a/retinoblastoma (RB), which are extensively used as biomarkers for identifying cellular senescence *in vivo* and *in vitro*. Although the respective contributions of the two effector routes to the initial growth arrest may differ depending on the type of stress, both may eventually be involved in senescence. Specific cyclin-dependent kinases (CDKs; CDK4, CDK6, and CDK2) phosphorylate RB1 and its family members, including RBL1 and RBL2 (Fig. 3).

Baixauli-Martín et al. demonstrated that the ischemic hemisphere showed considerably increased transcript expression of cell cycle arrest markers, including Cdk2a/ p16 INK4a, Cdkn1a/p21CIP1, and T/p53, compared to the non-ischemic hemisphere [205]. Similarly, p16 INK4a immunostaining showed that MCAO induced brain senescence in rats [207]. Torres-Querol et al. performed immunohistochemistry staining on postmortem brain samples from stroke donors with left middle cerebral artery infarction and found positive immunoreactivity for p16 and p21 in the adjacent ischemic core region [208]. Additionally, a significant increase in the number of p16-positive cells was observed in the infarcted region [208]. One of the best-studied biomarkers for senescence is p16, whereas other CDKISs, such as p21, p27, and p15, also accumulate in senescent cells and are crucial for preserving cell cycle arrest [211]. However, in other cell cycle arrest events, p53 is activated [212], and some nonsenescent cells express p16INK4A [213]. Therefore, various factors and traits must be quantified to identify cell cycle arrest linked to senescence in cerebral ischemic disorders.

**Table 3:** Rejuvenating therapy for ischemic stroke-related diseases, including immunomodulatory and anti-inflammatory therapy.

Senolytics	Stroke-related diseases	Senescent cell staining	Function	Ref.
Navitoclax (ABT-263)	Cerebral IR injury	SA-β-Gal staining	ABT263 eliminated senescent astrocytes (OGD/RA), reduced inflammation, decreased infarct volume, and improved neurological function.	[207]
	Cardiac IR injury	SA-β-Gal staining	ABT263 reduced the expression of SASP (IL-6, IL-11, IL-16, CCL22, MIP-3β, IP-10, fractalkine, eotaxin, and TGF-β), decreased infarct size and promoted angiogenesis, and eliminated senescent cells and improved cardiac function (improved left ventricular function, increased myocardial angiogenesis, and reduce scar size) after IR injury	[214]
	NVC	Sudan IV staining, SA-β-Gal staining	ABT263 eliminated senescent foam cells and reduced SASP components (Mmp3, Mmp13, Il1α, and TNF-α) and drivers of monocyte recruitment (MCP1 and VCAM1).	[215]
Dasatinib and Quercetin (D+Q)	Aged or atherosclerotic mice	telomere-associated foci (TAF’s); GFP mRNA in INK-ATTAC mice; nuclear γ-H2AX foci	D+Q reduced senescent cell load, DNA damage, vasodilatory function, NO signaling, and vascular calcification in aged mice.	[216]
	Endothelial senescence in atherosclerosis	SA-β-Gal staining	D+Q attenuated the senescence of HAECs and reduced apoptosis and ROS generation.	[217]
Rapamycin	Ischemic stroke	SA-β-Gal staining	Micro-dose Rapamycin maintained the nonproliferative state of cultured senescent human fibroblasts and prevented cell death caused by telomere dysfunction; in animal models, it reduced the area of acute ischemia-induced cerebral infarction, prolonged the lifespan of SHR-SP, prevented MPP+ and H_2_O_2_-induced neuronal death and extended the lifespan of cultured neurons, and reduced phosphorylated Ser2448 signals.	[218]
Celastrol	Vascular smooth muscle cell (VSMC) senescence	SA-β-Gal staining	Celastrol upregulated VSMC autophagy, reduced angiotensin (Ang) II and ROS, and inhibited PI3K/Akt/mTOR signaling pathway.	[219]
Metformin	VSMC senescence	SA-β-Gal staining	Metformin prevents vascular senescence. In senescent VSMC, metformin decreased SASP (p53, p21, and p16), upregulated autophagic flux, and attenuated lysosomal-associated membrane protein 1 (LAMP1) and tissue proteinase B (CTSB) levels.	[220]

IR, ischemia-reperfusion; NVC, neurovascular coupling; SA-β-Gal, senescence-associated β-galactosidase; TAF’s, telomere-associated foci; ROS, reactive oxygen species; HAECs, human aortic endothelial cells; IP-10, interferon gamma-induced protein 10; IL-6, interleukin-6; VSMC, Vascular smooth muscle cell; SHR-SP, stroke-prone spontaneously hypertensive rats; OGD/RA, oxygen-glucose deprivation/reoxygenation.

## 7. Interventions to Target Senescence in Stroke

Immunosenescence research shows potential in preventing and treating stroke and improving the health of aging populations (Table 3). Senescent cells cause immune cell dysfunction through increased DDRs, excessive ROS production, and SASP expression, which require potential strategies for drug treatment, including senolytics (eliminating senescent cells) or senostatics (suppressing the senescent phenotype) [221].

A new senolytic agent, navitoclax (ABT-263), causes apoptosis in senescent cells rather than in non-senescent cells by inhibiting the activity of anti-apoptotic BCL-2 family members [222]. Lim et al. demonstrated that the intravenous administration of ABT-263 effectively eliminated senescent cells in MCAO rats with brain IR injury, dramatically decreased infarct size, and improved neurological function [207]. Furthermore, ABT263 reduces the development of atherosclerosis in Ldlr^-/-^ mice by removing senescent foamy macrophages [215]. Tarantini et al. found that navitoclax treatment improved functional hyperemia in aged mice [223].

Quercetin (Q) is a flavonol that induces apoptosis in senescent cells by increasing the expression of SIRT1 and inhibits PI3K/mTOR signaling. Dasatinib (D), which is a tyrosine kinase inhibitor, induces senescent cell apoptosis by inhibiting the interaction of ephrin ligands with ephrin receptors [224]. Recent studies have demonstrated that quercetin reduces brain infarct size, neurological impairment, and BBB permeability by increasing the SIRT1 signaling pathway both *in vivo* and *in vitro* in tMCAO models [225]. However, senescent cell formation is a dynamic and ongoing process, and intermittent treatment with D+Q appears to be more effective than a single-dose treatment. Roos et al. found that combined intermittent D+Q treatment reduced the expression of senescent cell markers (TAF+ cells) in hyper-cholesterolemic mice and improved vasodilatory function in both senescent and atherosclerotic mice [216]. Similarly, D+Q treatment restores tight junction protein function in the brain microvascular endothelial cells and protects the brain barrier from age-related dysfunction [217]. Interestingly, another study revealed that the D+Q combination modulated intestinal aging and inflammation in aged mice [226].

Cells become senescent when both p53 and mTOR are activated; conversely, cells become quiescent when p53 is activated and mTOR is inhibited because mTOR regulates the selection between senescence and quiescence [227]. A microdose of rapamycin has been demonstrated to prolong senescence and limit neuronal death through cytoprotective activity, which reduces the area of acute ischemia-induced cerebral infarction and significantly extends the area in mice [218]. Celastrol is a plant-derived triterpene with neuroprotective properties against several types of brain injury [219]. Celastrol dramatically suppresses the mTOR signaling pathway, thereby reducing Ang II-induced intracellular ROS production and cellular senescence by activating autophagy, which contributes to the prevention and treatment of atherosclerosis [228]. Metformin, which is a frequently used diabetic therapy, also affects lifespan via AMPK activation [229]. Furthermore, metformin reduces brain infarct size and neuronal apoptosis via the AMPK signaling pathway in an ischemic rat model [230]. Metformin exerts protective effects against ischemic stroke by alleviating oxidative stress-mediated inflammation, promoting neurogenesis, and activating the PI3K/Akt1/JNK3/c-Jun pathway [231].

Novel mechanisms of inflammaging are likely to emerge in the near future, and such work will likely lead to potential therapeutic targets for age-related disorders. Therefore, swift and multidimensional interventions using novel preventative and therapeutic techniques are crucial.

## 8. Conclusion and Future Directions

Immunosenescence and inflammaging are rapidly becoming recognized as potential causes of stroke pathophysiology. Over time, the immune system undergoes dysregulation of complex molecular and cellular mechanisms that ultimately lead to altered functions and phenotypes. Therefore, understanding how individual cellular senescence types contribute to disease phenotypes will assist in developing more tailored and beneficial medicines to delay or prevent age-related disorders, such as stroke.

Although preclinical studies have demonstrated the effectiveness of age-specific interventions, clinical translation of these studies has been slow and even failed. Possible problems include the application of findings from studies in young mice to the treatment of older patients, which may obscure the potential of age-dependent candidate therapies. Moreover, most laboratory mice used for biomedical research are raised in clean and hygienic environments and thus are free of specific pathogens, resulting in an immune system that closely resembles the immune profile of newborn humans. In addition, aging patients who experienced stroke frequently have comorbidities, including hypertension, diabetes, cardiovascular disease, and atherosclerosis. Consequently, preclinical studies should consider the impact of these comorbidities on functional prognosis. With breakthroughs in genomics, transcriptomics, metabolomics, and other multi-omics studies, additional opportunities to fill these gaps will occur in the near future. First, the rise in transcriptomics has provided large amounts of data on the functional changes in age-associated immune cells after stroke, both in the acute and non-acute phases. Notably, non-enzymatic dissociation that occurs with transcriptional and translational inhibitors or at low temperatures minimizes the *in vitro* confounding (artifacts) of cell isolation techniques. Second, genetic ablation and pharmacological inhibition methods to remove senescent cells may help prevent and treat age-related diseases. Furthermore, distinguishing immunosenescence biomarkers, quantifying immunity, and setting normal reference ranges of immune cells among people of different ages must be performed to aid in the screening, prevention, and treatment of diseases, even at the subclinical stage.

Thus, future research should include prospective longitudinal studies in elderly patients who experienced stroke starting from the onset through symptom development to the recovery phase to characterize the exact contribution of changes in immune function. The long-term trajectory of changes influences the longitudinal recovery of homeostasis within the brain. Data from these elderly patients will serve as a basis for basic studies that can further elucidate the causality between age-related immune alterations and stroke pathophysiology. Moreover, the potential regulatory function of non-classical immune cells may have the same age-specific immunomodulatory role as immune cells. Understanding the complex interplay between immunosenescence and inflammaging enhances the targeting of new therapies to reduce the harmful effects of aging, maximizes the beneficial effects of the therapies, and may improve longevity.
